# Retrogene survival is not impacted by linkage relationships

**DOI:** 10.7717/peerj.12822

**Published:** 2022-01-24

**Authors:** Johnathan Lo, Heath Blackmon

**Affiliations:** Biology, Texas A & M University, College Station, Texas, United States

**Keywords:** Retrogene, Linkage, Recombination, Drosophila melanogaster

## Abstract

In retrogene evolution, the out-of-the-X pattern is the retroduplication of X-linked housekeeping genes to autosomes, hypothesized to be driven by meiotic sex chromosome inactivation during spermatogenesis. This pattern suggests that some retrogene survival is driven by selection on X-linkage. We asked if selection on linkage constitutes an important evolutionary force in retrogene survival, including for autosomal parents. Specifically, is there a correlation between retrogene survival and changes in linkage with parental gene networks? To answer this question, we compiled data on retrogenes in both *Homo sapiens* and *Drosophila melanogaster* and using Monte Carlo methods, we tested whether retrogenes exhibit significantly different linkage relationships than expected under a null assumption of uniform distribution in the genome. Overall, after excluding genes involved in the out-of-the-X pattern, no general pattern was found associating genetic linkage and retrogene survival. This demonstrates that selection on linkage may not represent an overarching force in retrogene survival. However, it remains possible that this type of selection still influences the survival of specific retrogenes.

## Introduction

RNA-mediated gene duplications (RMGDs) were first described in the early 1980s (Kaessmann, Vinckenbosch & Long, 2009). RMGDs emerge as a byproduct of reverse transcriptase (RT) activity, which itself is encoded and produced by Class I transposable elements (TEs) in the genome. RMGDs are created when the RT enzyme interacts with an mRNA to mediate its insertion into the genome (Casola & Betrán, 2017; Kaessmann, Vinckenbosch & Long, 2009). Because RMGD tends to omit regulatory elements and other context-specific sequences that help regulate expression, these retroposed gene copies, or retrocopies, were originally assumed to be non-functional. Indeed, the vast majority of retrocopies quickly accumulate mutations, making them non-functional; these are termed retropseudogenes. However, some retrocopies are conserved over long time periods; these functional retrocopies are termed retrogenes. Retrogenes can perform a variety of functions (*e.g.*, spermatogenesis, courtship behavior) that lead to their survival and fixation in a population (Kaessmann, Vinckenbosch & Long, 2009). Even non-protein coding retrogenes can be functional by contributing to gene regulation through production of fragmentary peptides and siRNAs, as demonstrated by the TP53 retroduplications discovered in elephants (Abegglen et al., 2015; Casola & Betrán, 2017; Sulak et al., 2016). From an evolutionary perspective, RMGD allows genes to explore a wider evolutionary space by removing constraints like intron-exon junctions and regulatory sequences (Kaessmann, Vinckenbosch & Long, 2009), and contributes to phenomena like exon shuffling and protein chimerism (Casola & Betrán, 2017; Kaessmann, Vinckenbosch & Long, 2009). Other retrogenes have been found to contribute to antiviral defenses, novel phenotypes in hormone-pheromone metabolism, brain development, and courtship behavior (Burki & Kaessmann, 2004; Dai et al., 2008; Sayah et al., 2004; Zhang et al., 2004). Retrogenes sometimes also supplant the function of parental genes (so-called “orphan” retrogenes) (Ciomborowska et al., 2013). While numerous individual retrogenes have been characterized, the nature of the evolutionary forces that lead to retrocopy survival are less clear.

One aspect of retrogene survival that has been left relatively unexplored is the impact of linkage. Because RMGD creates new gene copies with different linkage relationships, selection on linkage may influence the fate of retrocopies. Survival of retrocopies may be a path for mediating selection on linkage, in contrast to a direct modification of recombination rate. An RMGD-based model of linkage modification would thereby complement the modifier allele models proposed by Nei in 1967 and built upon in later decades by Feldman, Barton, Otto, and others (Feldman, Christiansen & Brooks, 1980; Nei, 1967; Otto & Barton, 1997; Otto & Barton, 2001; Otto & Michalakis, 1998). An RMGD-based model potentially overcomes several limitations of modifier allele models-for one, a modifier allele would not be able to resolve selection for tighter linkage between genes on different chromosomes, whereas RMGD would. Functional or structural limitations on modifier alleles may also restrict their capability for resolving selection on linkage, along with limitations on the environments where modifier allele mutations are predicted to fix (Feldman, Christiansen & Brooks, 1980; Otto & Barton, 2001; Otto & Michalakis, 1998). In this study, we investigate the possibility that selection on linkage influences retrogene survival using two empirical data sets. There are several existing lines of evidence that support the possibility of such a role.

Three previously characterized patterns of retrogene survival are the out-of-the-X pattern, retrogene replacement, and subfunctionalization, which are not mutually exclusive. The out-of-the-X pattern, documented in mammals and Drosophila, sees an excess of retrogenes originating from the X chromosome compared to what we would expect by chance. The expression of out-of-the-X retrogenes has been characterized as significantly male-biased, often with specific functions in spermatogenesis. In fact, a frameshift mutation in the out-of-the-X mouse gene *Utp14b* renders males completely deficient in spermatogenesis (Bradley et al., 2004). Retrogene replacement, on the other hand, sees retrogenes supersede the function of their parental genes, which are subsequently lost (Ciomborowska et al., 2013; Garstang & Ferrier, 2018). Twenty-five of these “orphan” retrogenes have been documented in humans; importantly, none of them exhibit testis-specific expression, although it is hypothesized that this had been the case originally (“out-of-the-testis”) (Garstang & Ferrier, 2018; Vinckenbosch, Dupanloup & Kaessmann, 2006). Additional examples of retrogene replacement have been found across diverse taxa. A striking example is found in all tetrapods, which share an instance of retrogene replacement in the dismantling of the ancestral Iroquois-Sowah genomic regulatory block (GRB) (Maeso et al., 2012). The ancestral GRB featured regulatory regions for Iroquois within Sowah’s introns; retrotransposition in the tetrapod lineage disentangled these genomic constraints while maintaining the developmental functions of both genes. Subfunctionalization, which divides ancestral gene function between parental and retrogene lineages, also encompasses some out-of-the-X retrogenes (Force et al., 1999; Hahn, 2009; Innan & Kondrashov, 2010; Kaessmann, 2010). This process is contrasted with neofunctionalization, where the duplicate acquires novel function(s), in line with Ohno (1970). In practice, these processes may be difficult to distinguish without in-depth comparative and functional analysis of expression patterns, and they potentially represent different stages of the same evolutionary trajectory (Casola & Betrán, 2017; He & Zhang, 2005). Still, distinct cases of both patterns have been observed in retrogene survival; a specific case of subfunctionalization is demonstrated in *CDC14Bretro*, whose protein product underwent adaptive relocation from microtubules to endoplasmic reticulum, while strong support exists for neofunctionalization in the fixation of *U2af1-rs1* in mouse (McCole et al., 2011; Rosso et al., 2008).

**Figure 1:**
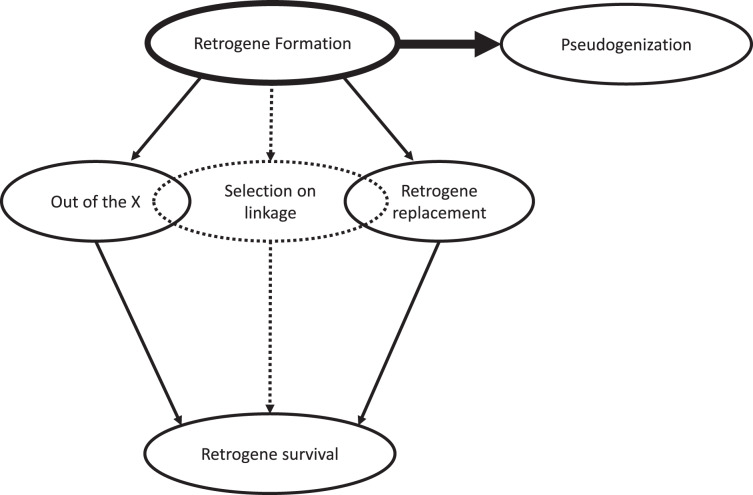
Diagram illustrating the fate of RMGDs. Most are pseudogenized, but various patterns areobserved in association with survival over evolutionary time-scales. Two existing patterns are the out-of-the-X pattern and retrogene replacement, both of which may represent special cases of selection onlinkage.

What drives the out-of-the X and retrogene replacement patterns? The leading hypothesis for the out-of-the-X pattern is that retrogenes allow previously X-linked housekeeping genes to escape meiotic sex chromosome inactivation during gametogenesis (Bai et al., 2007; Emerson et al., 2004; Otto & Barton, 1997). Conversely, haploid expression during spermatogenesis has also been shown to allow autosome-linked mutations to escape dominance effects experienced in somatic tissue (Raices, Otto & Vibranovski, 2019). Adaptive recessive mutations are exposed to selection through this mechanism and brought to fixation, among which may be newly formed retrocopies. In a more general sense then, the out-of-the-X phenomenon provides an example of how selection against maladaptive linkage patterns can promote retrogene survival, while haploid selection may be a mechanism through which retrogenes released from linkage constraints may gain a foothold in the population. As for retrogene replacement, there is no general hypothesis in the literature, but a case-by-case analysis yields two trends: either release from evolutionary constraints through loss of intron-exon junctions and regulatory regions or, as we propose, disentanglement from maladaptive linkage relationships (Casola & Betrán, 2017; Ciomborowska et al., 2013). The latter possibility is accentuated by the fact that, contrary to the expectation of relaxed selection on duplicates, orphan retrogenes show signs of elevated purifying selection (Ciomborowska et al., 2013). This elevated purifying selection is potentially the result of a release from Hill-Robertson interference impeding efficient selection on the parental gene. Finally, the leading hypothesis for subfunctionalization is the degeneration-duplication-complementation model, which posits that degeneration in regulatory regions of a gene increases fixation probability for duplicates (Force et al., 1999). However, the proximate reason for why degeneration in regulatory regions may promote fixation of gene duplicates is again due to maladaptive linkage relationships. Thus, for all of these cases, we propose that a more general mechanism underlying retrogene survival is selection against the existing linkage relationships of the parental gene (Fig. 1).

Here, we present a study into the influence of linkage on retrogene survival in humans and *Drosophila melanogaster*. We hypothesize that the changes in linkage resulting from retrocopy formation can contribute to the retrogene survival and fixation. Using data from RetrogeneDB, we created a structured dataset of retrogenes, parental genes, and parental network partners and their genomic coordinates (Kabza, Ciomborowska & Makałowska, 2014). Recombination maps were used to estimate genomic distances and construct sample statistics (Kong et al., 2002; Rezvoy et al., 2007). Corresponding reference distributions were constructed under the null assumption that retrogenes have a uniform random distribution across the genome independent of parental gene location. By testing our hypotheses against these reference distributions, we demonstrate a lack of significant association between changes in linkage and retrogene fixation. In general, retrogene fixation patterns match up well with expectations under the null hypothesis for the human and *D. melanogaster* retrogenes examined here; however, this study cannot completely discount the possibility of interaction between genetic distance and retrogene fixation and further investigation is warranted.

## Materials and Methods

### Data collection

Retrogene data was collected from http://yeti.amu.edu.pl/retrogenedb/ (accessed 3/21/21) (Kabza, Ciomborowska & Makałowska, 2014). Regulatory network data was collected from RegNetwork for humans, and JASPAR and REDfly for *D. melanogaster* (accessed 1/17/21) (Liu et al., 2015; Mathelier et al., 2013; Rivera et al., 2018). Assembly size, chromosome sizes, and genomic coordinates for each of the genes were collected from NCBI (human: GRCh38.p13, *D. melanogaster*: FB release 6). After removing retrocopies with missing information (IDs, protein-coding status, etc), a total of 4,426 retrocopies were found for humans, with 106 retrogenes. Retrocopies were paired with 1,431 parental genes with 809 unique network partners. In *D. melanogaster*, 82 retrocopies were found, 81 of which were retrogenes. These retrocopies were subsequently coupled with 64 parental genes with 109 unique network partners. Genetic distances were estimated using recombination maps for intrachromosomal parent-retrogene pairs; for interchromosomal pairs, distance was set to a default value of 0.5. (Kong et al., 2002; Rezvoy et al., 2007).

There are a number of significant differences between the human and *D. melanogaster* dataset. As mentioned, the human data contains many more pseudoretrogenes than the *D. melanogaster* data; this difference is likely an artifact of data collection and prior research directions rather than an indication of the true rate of pseudogenization. Most of the data on human pseudogenes originates from Ciomborowska et al. (2013), which extensively analyzes retrocopy content in humans; similar studies have not been conducted in *D. melanogaster*. Additionally, the density of regulatory networks differs between humans and *D. melanogaster*, with the latter exhibiting much denser and well-connected networks. Whether this is biological truth is a different question; however, for this study, we assume that the network data we have is an unbiased sample from the true relationships.

### Hypothesis testing

All analysis was done using R 3.9.1 in the RStudio 1.2.1335 IDE (R Core Team, 2017; RStudio Team, 2020). Packages used include *dplyr*, *rentrez*, *chromPlot*, *GenomicFeatures*, *MareyMap* and *reshape2* (Lawrence et al., 2013; Rezvoy et al., 2007; Verdugo & Orostica, 2019; Wickham, 2007; Wickham, 2016; Wickham et al., 2021). The reference distributions were generated using a Monte Carlo permutation method, with the null assumption that retrogenes are i.i.d. from a uniform random distribution over the genome. We had originally considered constructing the reference distributions by sampling retropseudogenes from our data; however, we decided against this for two reasons: firstly, the distribution of retropseudogenes observed in our data may not accurately reflect the true distribution of retrocopies at the time of retroduplication, and secondly, the distribution of retropseudogenes appears to be approximately uniform across the genome regardless (Fig. S1).

Samples of random retrogenes are simulated by drawing genomic coordinates from the above distribution. Relevant statistics (described later in the Results) are then calculated. For each of our tests, this process was iterated 1,000 times to produce an approximate reference distribution for the relevant statistic. Test statistics were then calculated using the empirical data and compared against the reference distribution using a two-tailed test (*α* = 0.05). All code and data used in this study are available *via* the author’s GitHub repository (https://github.com/johnathanlo/retrogenes).

## Results

### Testing data quality

Before proceeding with analysis, the quality of the dataset was assessed by searching for evidence of the well-documented out-of-the-X effect. As previously mentioned, this is a pattern in which more retrogenes originate from the X chromosome than expected through random chance. The statistic of interest is

$X_1 =\displaystyle \rm \{{{retrogenes \,from \,the \,{X} \,chromosome}\}}\cap \{{retrogenes \,not \,on \,the \,{X} \,chromosome}\}.$

Reference distribution was constructed by simulating random pairs of retrogenes and parental genes, then counting the number of pairs with a parental gene located on the X and a retrogene located on a different chromosome. In each realization of the Monte Carlo simulation, 106 retrogene/parental gene pairs were generated for humans, and 81 pairs for *D. melanogaster*, corresponding to each of the retrogenes in their respective datasets. This procedure was repeated 1,000 times to generate the null distribution. After calculating the test statistic from our data, significant results were found in both species, with *p* ∼ 0 (Fig. S1). A key assumption behind our testing procedure is the assumption that retrocopies and parental genes are distributed randomly in the genome.

### Retrogene distribution with respect to parental genes in humans

We wanted to test the linkage relationships for 106 retrogenes in humans and 81 retrogenes in *D. melanogaster*. To determine whether or not retrogenes are distributed independently of parental location, we analyzed the average genetic distance between a retrogene and its parental gene. The out-of-the-X effect was controlled for by removing all such parental genes/retrogenes from both datasets. Since our model posits a uniform random distribution of retrogenes independent of parental gene location, this should not bias our results on a subset of strictly autosomal parental genes.

The statistic is

**Figure 2:**
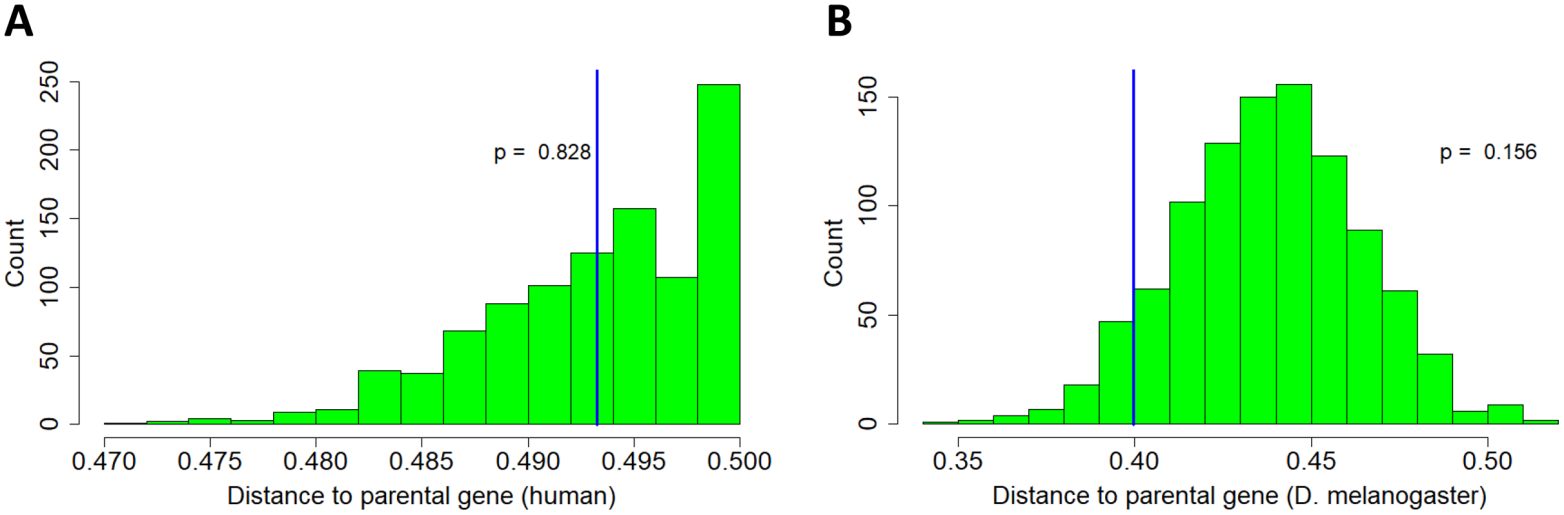
Tendency of retrogene movement away from parental chromosome. Histograms represent reference distribution obtained through 1,000-iteration Monte Carlo simulation. X-axis is distance in Morgans. Blue vertical line represents test statistic. (A) Human. (B) *D. melanogaster*

$X_2 = \displaystyle{\frac{\sum_{all\;b} dist(a,b)}\over{n}}$

where *a* is the parental gene, *b* is the retrogene, and *n* is the sample size (83 and 55 for humans and *D. melanogaster* respectively). The distance function is computed by applying recombination maps to the genomic coordinates of the parent/retrogene pair, with a default value of 0.5 for pairs on different chromosomes. The reference distribution was constructed as before by simulating a set of random parental genes and a corresponding set of random retrogenes and calculating the above statistic for 10,000 realizations. The test statistics were calculated from the data. No significant results were observed for either human or *D. melanogaster* (Fig. 2).

The above statistic weights each retrogene equally; however, there is heterogeneity in the number of retrogenes generated by each parental gene. To account for any bias resulting from this, we additionally test whether or not the average retrogene for each parental gene is more or less genetically distant than expected; in other words, we weight distances by parental genes instead. For each parental gene, we may calculate the statistic

$X_3 = \displaystyle\frac{\sum dist(a,x)}{|C|}$

where *a* is the parental gene and *x* ∈ *C*, where *C* is the set of retrocopies associated with *a*. *X*_3_ is therefore a function of the random variable *dist* (*a*, *x*). We define the mean over the population of parental genes as

$\mu = \displaystyle E(X_3).$

To obtain a distribution over the sample mean, we simulate the statistic *X*_3_ using Monte Carlo methods as before, then compute the estimate

**Figure 3:**
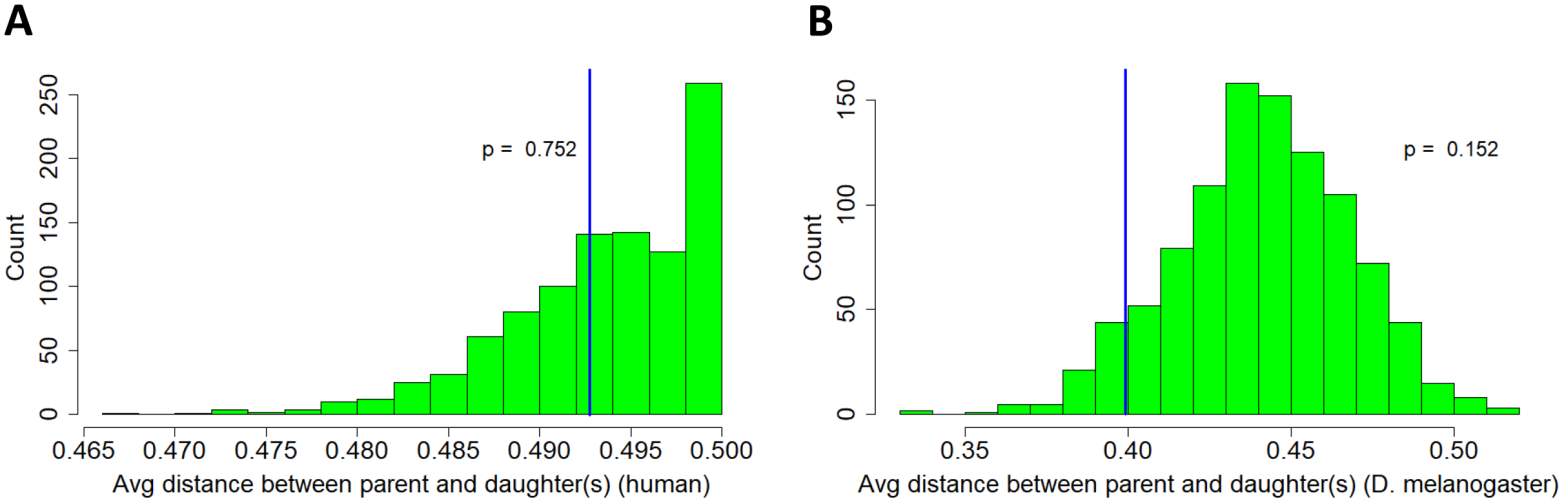
Movement of retrogenes relative to fixed parental genes. Histograms represent reference distribution obtained through 1,000-iteration Monte Carlo simulation. X-axis is distance in Morgans. Blue vertical line represents test statistic. (A) Human. (B) *D. melanogaster*

$\hat{\mu} = \displaystyle\frac{1}{n}\sum_{i=0}^n x_i$

in the usual way, where *x*_*i*_ assumed to be i.i.d. instances of *X*_3_ as defined above, and test at a significance level of *α* = 0.05. The results show that lack of significance persists regardless of whether genetic distance is weighted by retrogenes or by parental genes (Fig. 3). In other words, the average retrogene survival event is not influenced by linkage, and the average parental gene does not produce a distribution of retrogenes significantly different from random (null) expectation.

### Retrogene distribution relative to network partners

A final test was performed to assess whether retrogenes are randomly distributed with respect to their nearest network partners. Since we hypothesized that the linkage relationships of the parent may affect the fixation patterns of retrogenes, it is reasonable to extend this influence to network partners of the parent. Retrogene fixation may be influenced by gene regulatory network topology, since close proximity of a gene to network partners can ensure that certain combinations of alleles are inherited together as members of a co-adapted gene complex. Hence, we examine the relationship of retrogenes to the nearest network partner of parental genes, including the parental gene itself. We define a statistic describing the minimum distance between retrogenes and network partners as

$X_4 = \displaystyle\min \{ dist(a,x): x\in C\}$

where *a* is a retrogene, *x* is a network member/parental gene, *C* is the set of network partners of the parental gene and the parental gene, and *dist*(*a*, *x*) is the genetic distance between *a* and *x* as defined in the previous section; note *dist* (*a*, *x*) is a random variable, so *X*_4_ is random. Then we define the mean over the population

$\mu = \displaystyle E(X_4)$

and construct a distribution using Monte Carlo permutations as before. From the sample, we can then compute an estimate of the mean

**Figure 4:**
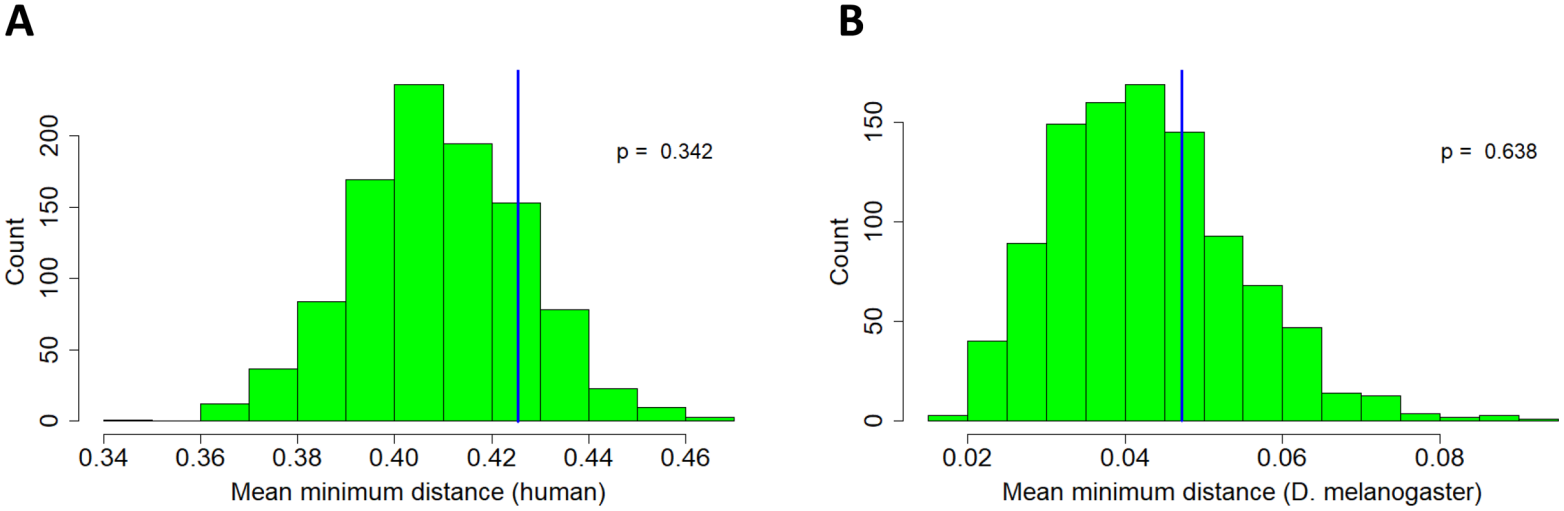
Movement of retrogenes relative to nearest network partners. Histograms represent reference distribution obtained through 1,000-iteration Monte Carlo simulation. X-axis is distance in Morgans. Blue vertical line represents test statistic. (A) Human. (B) *D. melanogaster*

$\hat{\mu} =\displaystyle\frac{1}{n}\sum_{i=0}^n x_i$

where we assume *x*_*i*_ i.i.d. samples from *X*_4_ and test at a significance level of *α* = 0.05. This test finds no significant deviation of the sample statistic from the expectation under the null for either humans or *D. melanogaster* (Fig. 4). In other words, the distribution of retrogenes in the genome does not seem to be influenced by proximity to parental network partners.

## Discussion

The dynamics of retrogene fixation have been a wellspring of fascinating evolutionary tales. While a number of specific evolutionary patterns have been discerned, such as the out-of-the-X phenomenon and retrogene replacement, retrogene evolution has not yet been broadly characterized (Abegglen et al., 2015; Ciomborowska et al., 2013; Emerson et al., 2004). One possible force that could play a role in shaping retrogene fixation patterns is their linkage relationships. Specifically, in copying a parental gene to a new location, new linkage is formed and old linkage is lost. For alleles experiencing clonal interference or Hill-Robertson interference, RMGD provides a concise mechanism for mediating those selective forces. This mechanism can manifest either when retrogene survival helps alleviate selection against Hill-Robertson interference, or when retrogene survival is selected for by clonal interference (Roze & Barton, 2006). This mechanism also provides an interesting alternative for modifying recombination rates compared to the traditional modifier allele model. We tested this possibility to determine if retrogene fixation is influenced by features of the genetic distance landscape. Overall, no support was found for this possibility.

### Linkage patterns do not play significant role in retrogene fixation

None of our tests exhibit any significant association between changes in linkage and retrogene fixation in either humans or *D. melanogaster*. In fact, the results markedly conform to our null expectation of uniformly random distribution of retrogenes across the genome. In other words, given that a retrogene fixes, it does not have any tendency to be closer or further to either its parental gene or the network partners of its parental genes.

### Linkage with nearest network partners does not affect retrogene fixation

The relationship between retrogenes and network partners was also investigated. We investigated the linkage relationship between retrogenes and their nearest network partner specifically. No significant correlations were observed in either humans or *D. melanogaster*. These findings do not exclude the possibility of a broader effect involving network partners. The nearest network partner is insufficient to fully characterize the topology of an entire regulatory network, and it remains plausible that the overall topology of the network is correlated with changes in linkage from parental gene to retrogene.

### Concluding Remarks

This study provides theoretical background and a preliminary investigation into a novel hypothesis regarding retrogene evolution. While these results do not indicate a significant role for linkage in determining retrogene fixation, several limitations and confounding factors provide basis for further investigation. One confounding factor is that variation in selection on linkage (*e.g.*, selection for increased linkage in some lineages *vs* selection for decreased linkage in others) may mask signals from detection by our methods. Indeed, we know that in mammals and Drosophila, the out-of-the-X pattern is a definitive example of selection against linkage, while other studies have demonstrated that proximity may sometimes be selected for to derive the benefits of nearby regulatory regions or open chromatin formations (Bai, Casola & Betrán, 2008; Loppin et al., 2005). Additionally, even though we find no consistent pattern, selection on linkage may still have been key to the survival of a subset of retrogenes. Our study assumes that retrogenes are i.i.d. with respect to genetic distances, a simplification that may not hold up in reality. To uncover effects of linkage on the level of individual genes would require in-depth functional and comparative analysis of suspected retrogene/parental gene pairs similar to work on the out-of-the-X retrogenes. The analysis is of course limited also by constraints related to available data; data from diverse taxa may be necessary to sufficiently illuminate the role of linkage in retrogene survival.

Finally, it remains interesting to ask whether or not selection on linkage can be mediated through retrogene fixation, much like the argument for the fixation of modifier alleles. The primary obstacle to such a study is finding a sample of genes that have experienced selection on their linkage relationships at some point in the past. With such a sample, we can ask whether or not they produce retrogenes at a greater than normal rate. Constructing such a sample with any certitude may seem like a daunting task, but one that may be amenable through experimental evolution techniques.

There are plausible reasons for why selection on linkage may play a role in retrogene fixation only rarely. Previous work has strongly emphasized neofunctionalization as an outcome of retrogene fixation, which would make such retrogenes less likely to interact with the parental gene or its network partners (Casola & Betrán, 2017; He & Zhang, 2005). Our work suggests exactly this: the observed lack of correlation between parental gene networks and retrogenes may indicate that retrogenes typically occupy different regulatory networks and fulfill different functions when compared to their parents. This phenomenon may also be diminished by limitations on the expression of new retrocopies. New retrocopies require expression to be selected for, and since they do not typically carry regulatory elements with them, they may not achieve consistent or appreciable levels of expression, which prevents selection from acting. The primary venue for new retrocopies to achieve high levels of expression is during promiscuous expression, as in the thymus and testes, or during haploid expression during spermatogenesis (Casola & Betrán, 2017; Raices, Otto & Vibranovski, 2019). Then, retrocopies may only be selected for if their expression conveys fitness benefits under these particular contexts, and retrocopies that do not provide any effect or benefit during spermatogenesis would thus be less likely to experience selection.

As genomic sequencing and analysis tools improve, analysis of retrogene evolutionary dynamics in other species will become feasible. Further investigation of the hypotheses presented here might be best served by data from deer and muntjac (Mudd et al., 2020; Yang et al., 1997). Their shared evolutionary history makes comparisons between findings between species feasible; additionally, these lineages have undergone multiple chromosome fusions and fissions in recent evolutionary time, which provide a backdrop of changes in linkage that make some form of selection on linkage nearly inevitable. Another potentially fruitful line of inquiry would be to ask if specific classes of parental genes and retrogenes are more influenced by selection on linkage than others. For example, recent work has found autosomal pairs of parental genes and retrogenes that exhibit complementarity of expression in the testes, which may appear to be a more likely scenario for selection on linkage (Casola & Betrán, 2017). Both of the above present reasonable directions for future work in this area.

## Supplemental Information

10.7717/peerj.12822/supp-1 Supplemental Information 1 Plots demonstrating distributions and data checks. (A) Distribution of retropseudogenes across the human genome. (B) Distribution of parental genes across the human genome. (C, D) Testing the out-of-the-X hypothesis in humans and *D. melanogaster* respectively. Each histogram shows the number of retrogene parents on the X chromosome expected under the null hypothesis. The blue line indicates the number of retrogene parents that are observed in the empirical dataset.

## Additional Information and Declarations

### Data Availability

The following information was supplied regarding data availability: All code and data used in this study are available at GitHub: https://github.com/johnathanlo/retrogenes.
