# Supplementary figures and images for: Retrogene survival is not impacted by linkage relationships
Source: PeerJ. 2022 Jan 24;10:e12822. doi: 10.7717/peerj.12822 (PMC8793726; doi:10.7717/peerj.12822)

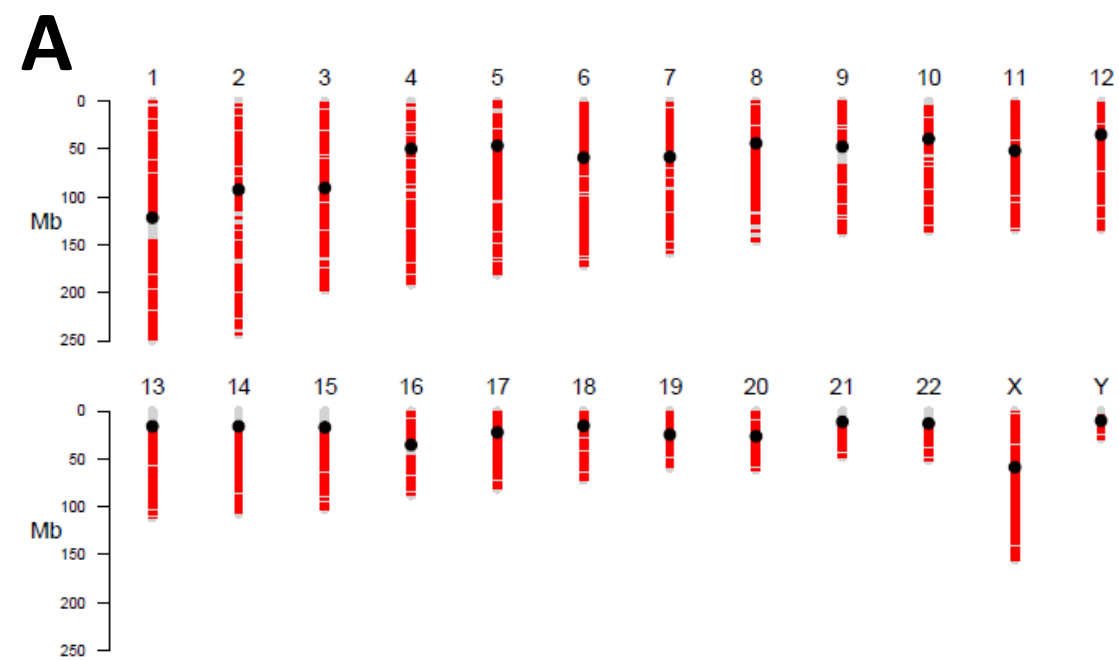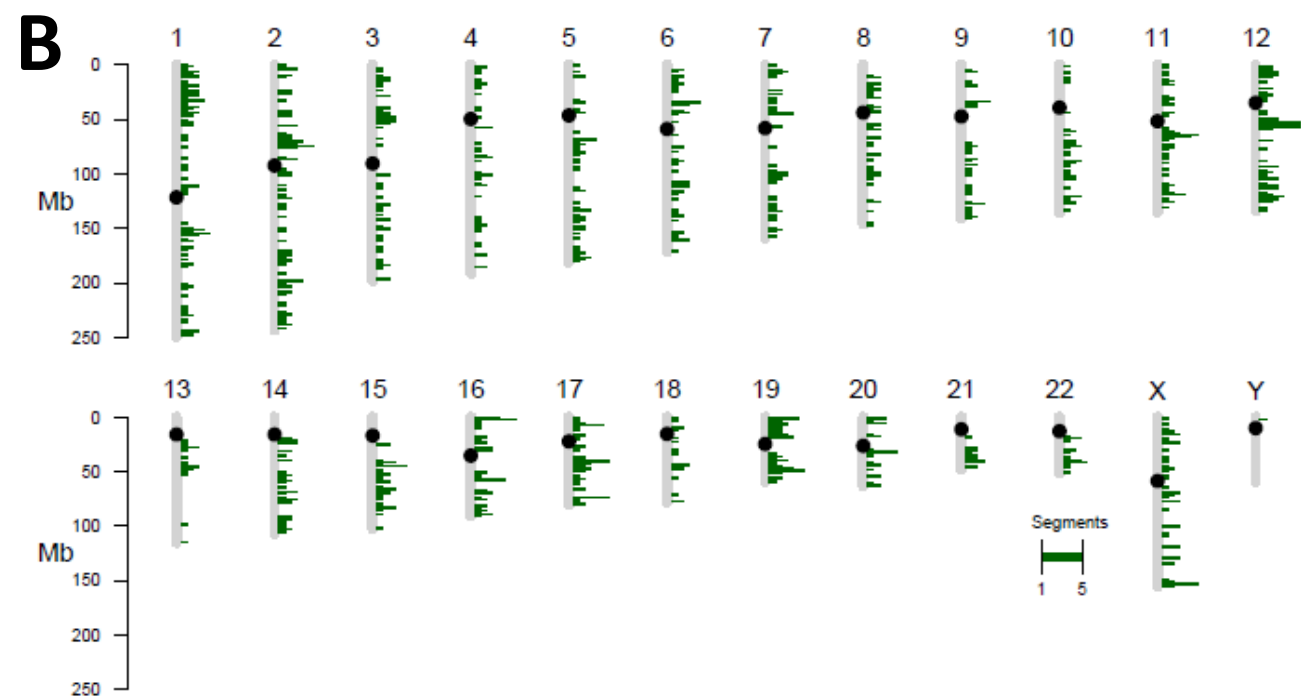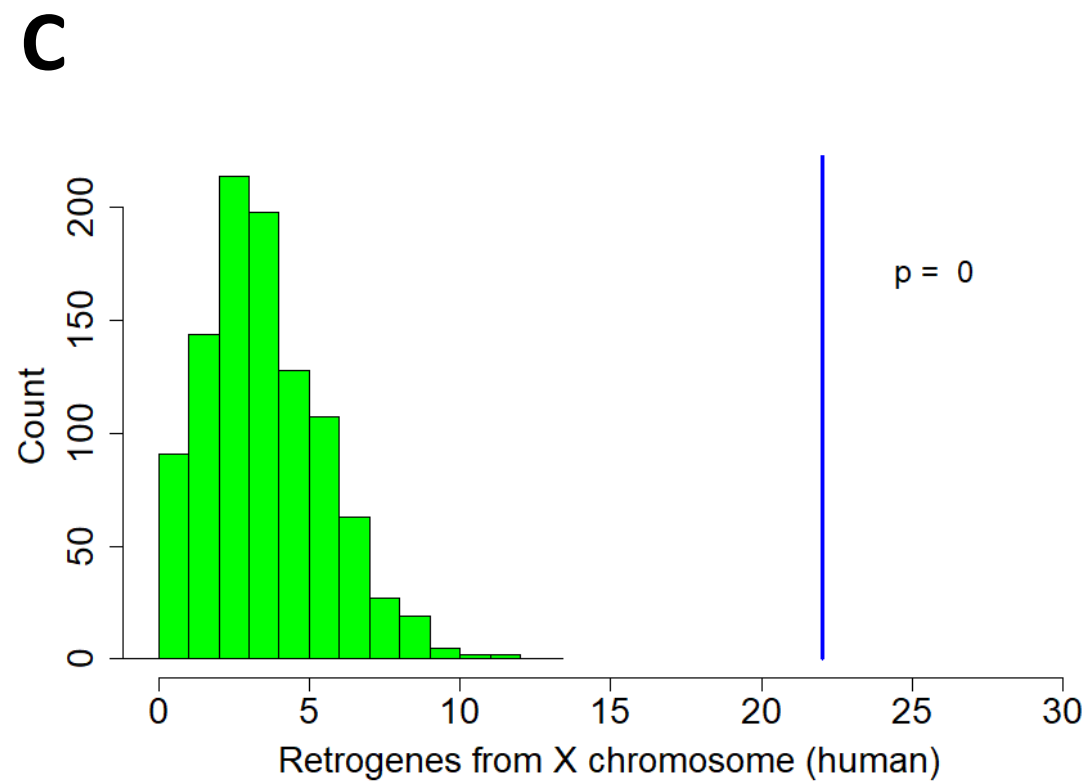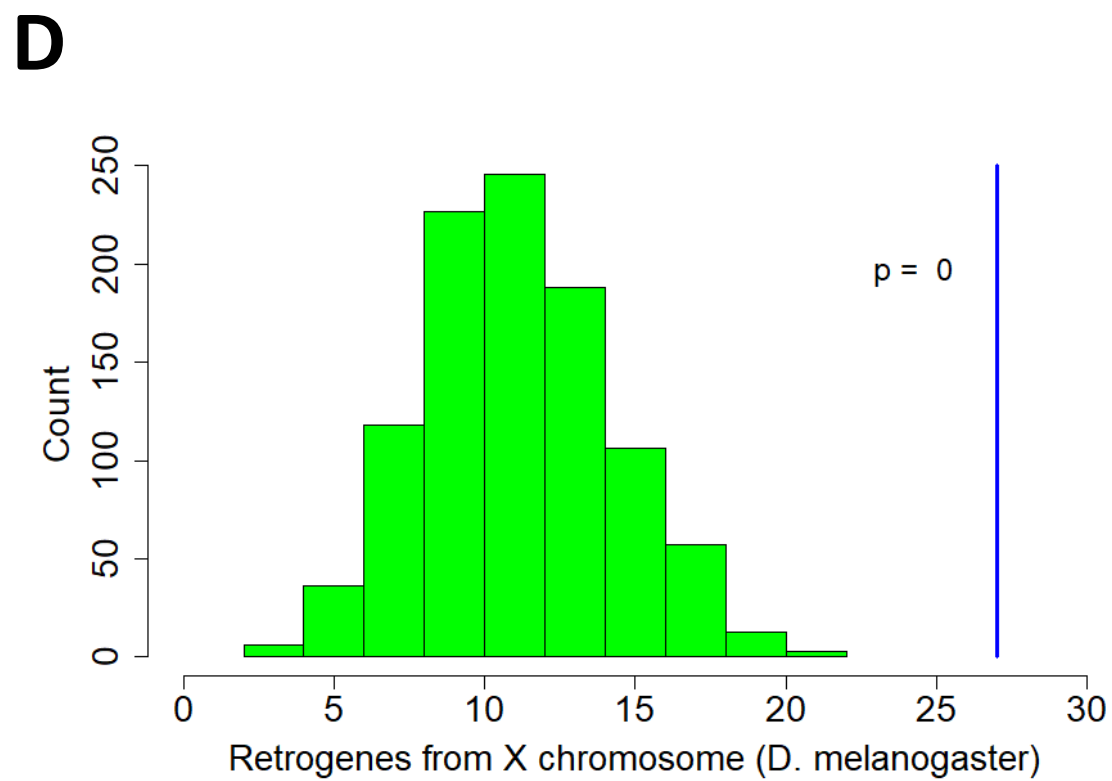

Supplement: Supplemental Information 1 — (A) Distribution of retropseudogenes across the human genome. (B) Distribution of parental genes across the human genome. (C, D) Testing the out-of-the-X hypothesis in humans and D. melanogaster respectively. Each histogram shows the number of retrogene parents on the X chromosome expected under the null hypothesis. The blue line indicates the number of retrogene parents that are observed in the empirical dataset. [file peerj-10-12822-s001.pdf]
